# New Genera and Species of the Family Throscidae (Coleoptera: Elateroidea) in Mid-Cretaceous Burmese Amber

**DOI:** 10.3390/insects12010063

**Published:** 2021-01-12

**Authors:** Yan-Da Li, Di-Ying Huang, Chen-Yang Cai

**Affiliations:** 1 State Key Laboratory of Palaeobiology and Stratigraphy, Nanjing Institute of Geology and Palaeontology and Center for Excellence in Life and Paleoenvironment, Chinese Academy of Sciences, Nanjing 210008, China; ydli@pku.edu.cn (Y.-D.L.); dyhuang@nigpas.ac.cn (D.-Y.H.); 2 School of Life Sciences, Peking University, Beijing 100871, China; 3 School of Earth Sciences, University of Bristol, Life Sciences Building, Tyndall Avenue, Bristol BS8 1TQ, UK

**Keywords:** Throscidae, palaeodiversity, Mesozoic, Burmese amber

## Abstract

**Simple Summary:**

Throscidae is a relatively small lineage in the beetle superfamily Elateroidea. The Mesozoic fossil records of this family are sparse. Here we describe three new throscid species found in mid-Cretaceous Burmese amber, all represented by well-preserved specimens. These newly discovered species suggest that Throscidae had a high diversity in the Cretaceous.

**Abstract:**

*Captopus depressiceps***gen. et sp. nov.**, *Electrothroscus yanpingae***gen. et sp. nov.** and *Pseudopactopus robustus* **gen. et sp. nov.** are reported from the mid-Cretaceous Burmese amber. These new findings greatly extend the Mesozoic diversity of Throscidae, which implies a high degree of morphological disparity for this family in the Cretaceous.

## 1. Introduction

The family Throscidae is one of the relatively small lineages of elateroids, with five extant genera and about 150 extant species [1]. Throscids are characterized by their unusual antennal grooves running by the notosternal suture and extending posteriolaterad along the hind margins of hypomera, which could also be a possible apomorphy of this family [2,3]. Historically, Throscidae also included genera such as *Drapetes* Dejean and *Lissomus* Dalman (e.g., [4,5,6]), which were later moved into Elateridae, as supported by both morphological and molecular studies [7,8,9]. Throscidae appears to be closely related to the elateroid families Eucnemidae and Cerophytidae, but the relationship among them is far from being settled. Analytical phylogenetic studies have suggested Throscidae to be the sister group of Lissominae + Thylacosterninae [10], Eucnemidae [11], Cerophytidae [9], or Eucnemidae + Cerophytidae [8,12], or an independent lineage [9].

Most fossils of Throscidae were discovered in amber deposits, including Lebanese amber [2] and Burmese amber [3,13] of the Cretaceous, and Oise amber [14] and Baltic amber [3,15] of the Oligocene to Eocene. Despite the great diversity of fossil insects preserved in the mid-Cretaceous Burmese amber [16,17], only two throscid species has been reported in this material to date [3,13]. Here, we report another three members of Throscidae from Burmese amber, which greatly enrich our knowledge on the early diversity of this family.

## 2. Materials and Methods

The Burmese amber specimens studied here are derived from amber mines near Noije Bum Village (26°20′ N, 96°36′ E), Hukawng Valley, Kachin State, northern Myanmar. The specimens are deposited in the Nanjing Institute of Geology and Palaeontology, Chinese Academy of Sciences (NIGP), Nanjing, China. The amber piece was trimmed with a small table saw, ground with emery papers of different grit sizes, and finally polished with polishing powder. Photographs under incident light were taken with a Discovery V20 stereo microscope (Zeiss, Jena, Germany). Widefield fluorescence images were captured with the Zeiss Axio Imager 2 light microscope combined with a fluorescence imaging system. Confocal images were obtained with a Zeiss LSM710 confocal laser scanning microscope. Images under incident light or widefield fluorescence were automatically stacked in Helicon Focus 7.0.2 or Zerene Stacker 1.04. Confocal images were automatically stacked and colour-coded in ZEN 2011 or ZEN 2.3 (Blue Edition), or manually stacked in Adobe Photoshop CC. In the multicoloured images, the colours were coded based on z-depth. Images were further processed in Adobe Photoshop CC to enhance contrast. This published work and the nomenclatural acts have been registered in ZooBank, the official registry of Zoological Nomenclature. The LSID for this publication is urn:lsid:zoobank.org:pub:4797AF94-8912-4DCA-B375-A7D25F16B2A6.

## 3. Systematic Palaeontology

Order Coleoptera Linnaeus, 1758

Suborder Polyphaga Emery, 1886

Superfamily Elateroidea Leach, 1815

Family Throscidae Laporte, 1840

 

**Diagnosis.** Antennae 11-segmented, with a distinct club; antennomere 1 attached to antennomere 2 apically. Prothoracic antennal grooves running along the notosternal suture and extending posterolaterally along the hind margins of the hypomera. Prosternal process apically acute, fitting into mesoventral cavity. Metacoxal plates medially extending posteriorly. Abdomen with five connate ventrites.

 

**Genus*****Captopus*** Li & Cai **gen. nov.**

 

**LSID.** urn:lsid:zoobank.org:act:CB5975DC-1566-406B-88C1-B70512D32B40

**Type species.** *Captopus depressiceps* **sp. nov.**

**Etymology.** The generic name is an anagram of “*Pactopus*”, another genus in Throscidae. The name is masculine in gender.

**Diagnosis.** Frons without carina, but with a deep cavity on both sides. Eyes large. Lateral pronotal ridge incomplete. Prosternum with subparallel prosternal carinae. Metaventrite with well-developed mesotarsal grooves. Abdomen with metatarsal grooves extending beyond the posterior margin of the third ventrite.

**Remarks.** The new genus *Captopus* is similar to the extant genus *Pactopus* Horn and the extinct genus *Tyrannothroscus* Muona in having both well-developed mesotarsal and metatarsal grooves. *Captopus* shows some crucial differences from other known extant or extinct species in *Pactopus*. For example, the metatarsal grooves of *Pactopus* do not extend beyond the posterior edge of ventrite 3, while in *Captopus*, the metatarsal grooves extend well beyond ventrite 3 and reach the middle of ventrite 4. More importantly, there is a deep cavity on both sides of frons in *Captopus*, formed by the expansion of the groove around the ridge bordering antennal insertion. This feature is unique among all known extant and extinct throscids. *Captopus* differs from *Tyrannothroscus* additionally in having no supraocular ridges, and lateral pronotal ridge incomplete.

 

**Figure 1:**
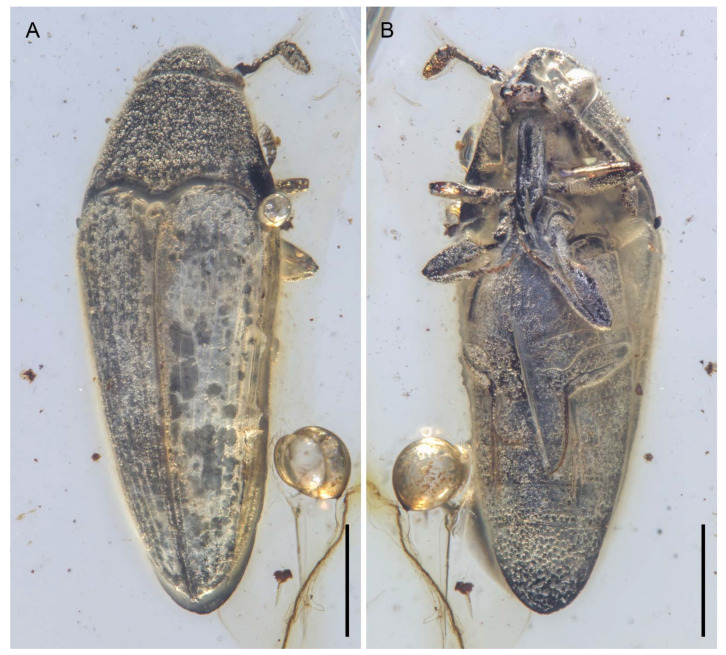
General habitus of *Captopus depressiceps* **gen. et sp. nov.**, holotype, NIGP173915, under incident light. (**A**) Dorsal view. (**B**) Ventral view. Scale bars: 500 μm.

**Figure 2:**
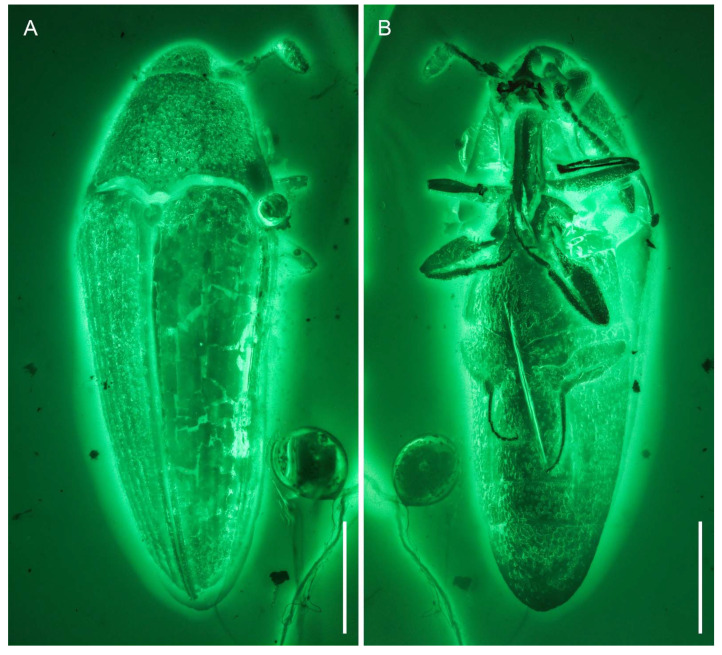
General habitus of *Captopus depressiceps* **gen. et sp. nov.**, holotype, NIGP173915, under widefield fluorescence. (**A**) Dorsal view. (**B**) Ventral view. Scale bars: 500 μm.

**Figure 3:**
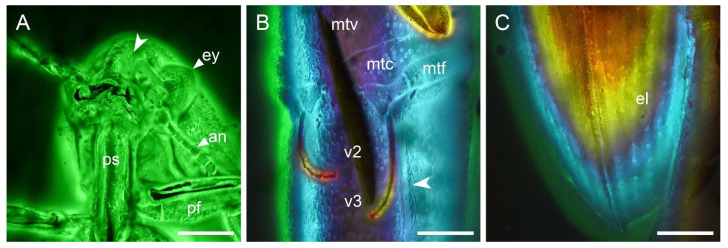
Details of *Captopus depressiceps* **gen. et sp. nov.**, holotype, NIGP173915, under confocal microscopy, with depth colour coding in (**B**,**C**). (**A**) Head and prothorax, ventral view, showing the deep cavity on frons (arrowhead). (**B**) Hind legs and abdomen, ventral view, showing the metatarsal groove (arrowhead). (**C**) Apex of elytra, dorsal view. Abbreviations: an, antenna; el, elytron; ey, compound eye; mtc, metacoxa; mtf, metafemur; mtv, metaventrite; pf, profemur; ps, prosternum; v2–3, ventrites 2–3. Scale bars: 200 μm.

*Captopus depressiceps* Li & Cai **sp. nov.** (Figure 1, Figure 2 and Figure 3).

 

**LSID.** urn:lsid:zoobank.org:act:4D81D22F-D7B1-4BD3-B4AB-03B594624094

**Etymology.** The specific name is derived from the Latin “*depressus*”, sunken, and “*caput*”, head, referring to the deep cavities on its head.

**Material.** Holotype, NIGP173915, an exceptionally well-preserved specimen.

**Locality and horizon.** Amber mine located near Noije Bum Village, Tanai Township, Myitkyina District, Kachin State, Myanmar; unnamed horizon, mid-Cretaceous, Upper Albian to Lower Cenomanian [18,19].

**Diagnosis.** As for the genus.

**Description.** Body elongate, length 2.5 mm, width 0.9 mm.

Head (Figure 3A) transverse; frons without median carina, but with a deep cavity on both sides formed by the expansion of the sulcus around the ridge semi-encircling antennal insertion. Eyes large, moderately emarginate, finely facetted, without interfacetal setae. Antennae with 11 antennomeres; antennomere 1 (scape) large, longer than wide, attached to antennomere 2 apically; antennomere 2 subrectangular, shorter and narrower than scape; antennomere 3 thinner than antennomere 2; terminal three antennomeres enlarged, forming a club.

Pronotal disc about 1.6 times as wide as long along the middle; sides not sinuate, converging anteriorly; posterior angles strongly acute and produced posteriorly; posterior edge bisinuate, with an additional slight emargination in the middle fitting against scutellum; lateral pronotal ridge incomplete. Scutellum polygonal. Elytra widest behind the base, about 2.1 times as long as wide combined; elytral surface with punctate striae (Figure 3C).

Prosternum subtrapezoidal; prosternal carinae almost parallel; prosternal process apically acute, fitting into mesoventral cavity. Metaventrite without discrimen; mesotarsal grooves well-developed (Figure 3B). Mesocoxae round; metacoxal plates medially extending posteriorly. Tarsal formula 5-5-5; pretarsal claws simple.

Abdomen with five connate ventrites; ventrite 5 twice as long as ventrite 4; metatarsal grooves well-developed, arcuate, reaching the middle of ventrite 4.

 

**Genus*****Electrothroscus*** Li & Cai **gen. nov.**

 

**LSID.** urn:lsid:zoobank.org:act:8A15D241-02F9-4A9C-8144-94944D2E51DD

**Type species.** *Electrothroscus yanpingae* **sp. nov.**

**Etymology.** The generic name is derived from the Greek noun “*elektron*”, amber, and the generic name *Throscus* Latreille, a junior synonym of *Trixagus* Kugelann. The name is masculine in gender.

**Diagnosis.** Frons without modifications. Eyes large. Prosternum with subparallel prosternal carinae. Metaventrite without mesotarsal grooves. Abdomen without metatarsal grooves.

**Remarks.** In Throscidae, only *Pseudothroscus* Muona is known to lack median head carina and both tarsal grooves [3]. *Pseudothroscus* is also characterized by its nonparallel prosternal carinae [13]. However, the prosternal carinae of *Electrothroscus* are subparallel, which is similar to most Throscidae.

 

**Figure 4:**
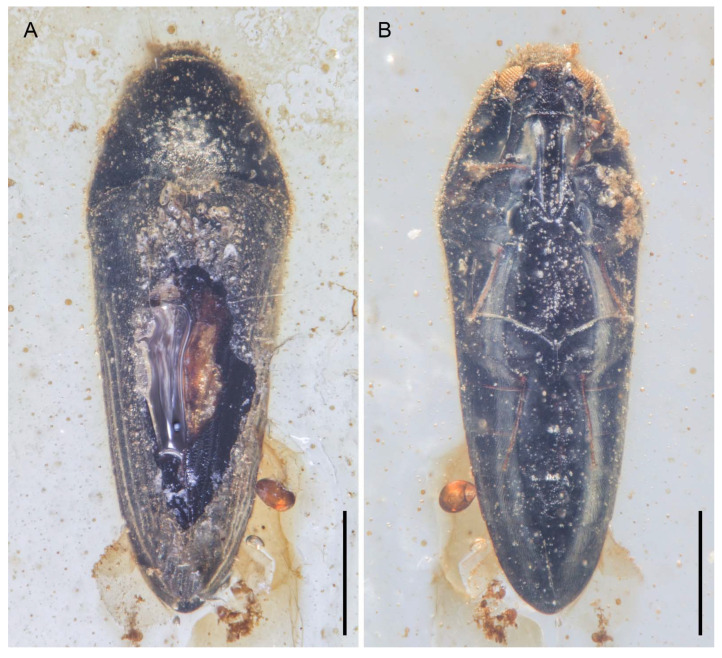
General habitus of *Electrothroscus yanpingae* **gen. et sp. nov.**, holotype, NIGP173916, under incident light. (**A**) Dorsal view. (**B**) Ventral view. Scale bars: 500 μm.

**Figure 5:**
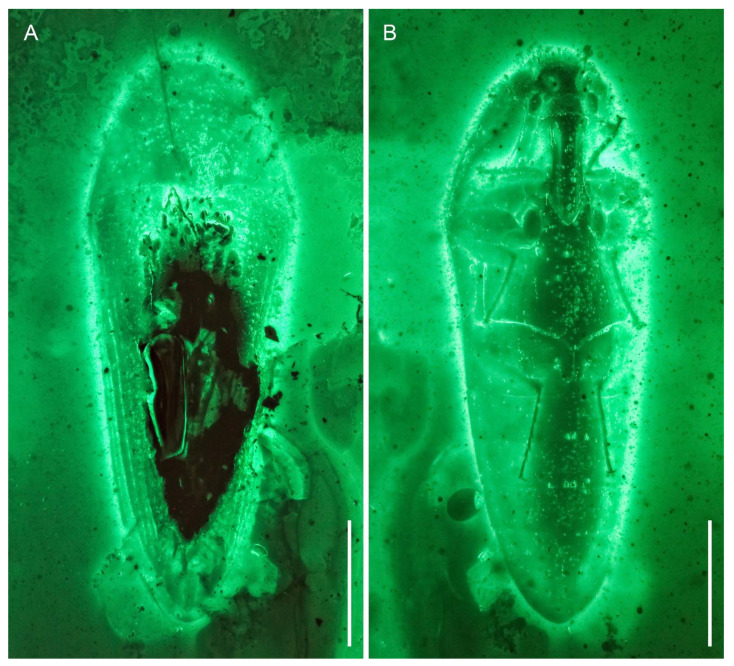
General habitus of *Electrothroscus yanpingae* **gen. et sp. nov.**, holotype, NIGP173916, under widefield fluorescence. (**A**) Dorsal view. (**B**) Ventral view. Scale bars: 500 μm.

**Figure 6:**
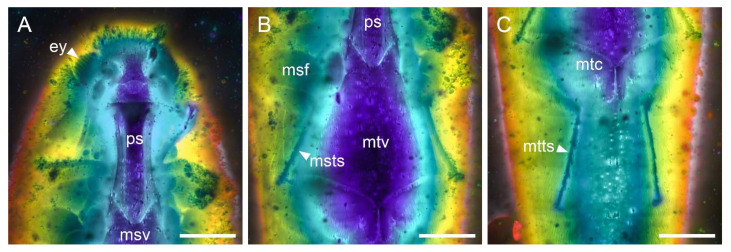
Details of *Electrothroscus yanpingae* **gen. et sp. nov.**, holotype, NIGP173916, under confocal microscopy, with depth colour coding. (**A**) Head and prothorax, ventral view. (**B**) Middle legs and metaventrite, ventral view. (**C**) Hind legs and abdomen, ventral view. Abbreviations: ey, compound eye; msf, mesofemur; msts, mesotarsus; msv, mesoventrite; mtc, metacoxa; mtts, metatarsus; mtv, metaventrite; ps, prosternum. Scale bars: 200 μm.

*Electrothroscus yanpingae* Li & Cai **sp. nov.** (Figure 4, Figure 5 and Figure 6).

 

**LSID.** urn:lsid:zoobank.org:act:76502D09-035D-4F20-9A0A-CA568096CF45

**Etymology.** The specific name is after Prof. Yan-Ping Guo (Beijing Normal University), an evolutionary botanist.

**Material.** Holotype, NIGP173916, a well-preserved specimen with elytra partially polished away.

**Locality and horizon.** Amber mine located near Noije Bum Village, Tanai Township, Myitkyina District, Kachin State, Myanmar; unnamed horizon, mid-Cretaceous, Upper Albian to Lower Cenomanian [18,19].

**Diagnosis.** As for the genus.

**Description.** Body elongate, length 2.3 mm, width 0.8 mm.

Head transverse (Figure 6A); frons without median carina or other modifications. Eyes large, slightly emarginate, finely facetted, without interfacetal setae. Antennae not properly visible; antennomere 1 attached to antennomere 2 apically; terminal three(?) antennomeres enlarged, forming a club.

Pronotal disc about 1.6 times as wide as long along the middle; sides not sinuate, converging anteriorly; posterior angles strongly acute and produced posteriorly; posterior edge bisinuate. Elytra widest behind the base, about 2.2 times as long as wide combined.

Prosternum subtrapezoidal; prosternal carinae almost parallel; prosternal process slightly narrowed in middle, apically acute, fitting into mesoventral cavity. Metaventrite without discrimen; mesotarsal grooves absent (Figure 6B). Mesocoxae round; metacoxal plates medially extending posteriorly. Tarsal formula 5-5-5; pretarsal claws simple.

Abdomen with five connate ventrites; ventrite 5 twice as long as ventrite 4; metatarsal grooves or impressions absent (Figure 6C).

 

**Genus*****Pseudopactopus*** Li & Cai **gen. nov.**

 

**LSID.** urn:lsid:zoobank.org:act:8CA0EA80-040B-4924-AF10-F9D0F6453D1C

**Type species.** *Pseudopactopus robustus* **sp. nov.**

**Etymology.** The generic name is derived from the Greek “*pseudes*”, false, and the generic name *Pactopus* LeConte. The name is masculine in gender.

**Diagnosis.** Body wide (length/width ratio ~2.1). Frons without modifications. Eyes large. Lateral pronotal ridge incomplete. Prosternum with subparallel prosternal carinae. Protibiae simple. Metaventrite with well-developed mesotarsal grooves. Abdomen with metatarsal grooves reaching or almost reaching the posterior margin of the third ventrite.

**Remarks.** The new genus *Pseudopactopus* possesses well-developed mesotarsal and metatarsal grooves, which, together with its unmodified head, link the new genus with *Pactopus*. However, the protibiae of *Pseudopactopus* are simple (Figure 8b), while protibiae are enlarged apically and with tarsal grooves in *Pactopus* (Figure 12 in [3]). Such simple protibiae are known previously only in *Pseudothroscus*, *Tyrannothroscus*, *Potergosoma* Kovalev & Kirejtshuk, *Rhomboaspis* Kovalev & Kirejtshuk, and *Trixagosoma* Li et al. [20].

Additional characters also differentiate *Pseudopactopus* from *Pactopus*. The body shape of *Pseudopactopus robustus* is broader than all other known extant and extinct *Pactopus* species. The type specimens of *Pactopus burmensis* Muona and *Pactopus americanus* Wickham have a length/width ratio of about 2.4–2.5, and *Pactopus horni* LeConte, *Pactopus fasolti* Muona and *Pactopus fafneri* Muona are even much narrower [3]. By contrast, the width of *Pseudopactopus robustus* almost reaches half of its length. The sides of pronotal disc are more or less sinuate in *P. horni* [21], and also possibly sinuate in *P. americanus* (Plate VI, Figure 10 in [22]), whereas *Pseudopactopus* does not have such sinuate sides of pronotal disc. Similar to *P. horni* and *P. americanus*, the metatarsal grooves of *Pseudopactopus* reaches the posterior margin of the third ventrite. However, in *P. fasolti* and *P. fafneri*, the metatarsal grooves do not even reach the posterior half of the third ventrite (Figures 13 and 15 in [15]).

 

**Figure 7:**
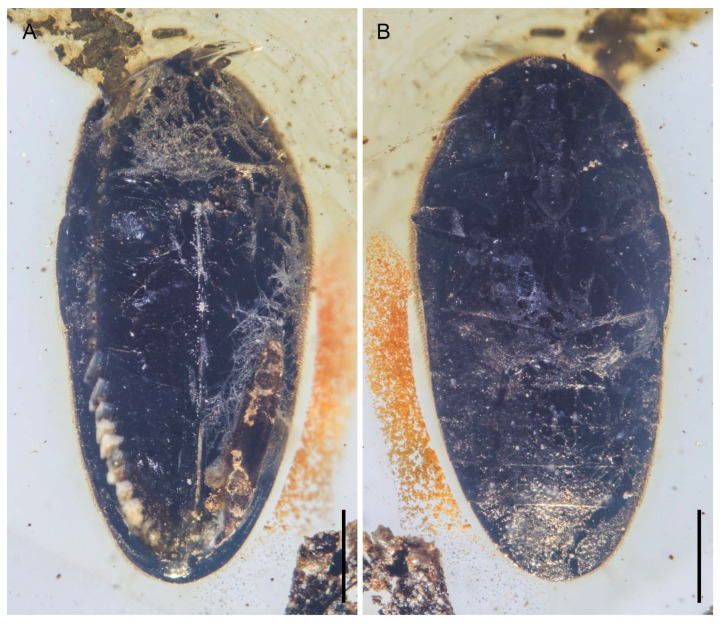
General habitus of *Pseudopactopus robustus* **gen. et sp. nov.**, holotype, NIGP173917, under incident light. (**A**) Dorsal view. (**B**) Ventral view. Scale bars: 500 μm.

**Figure 8:**
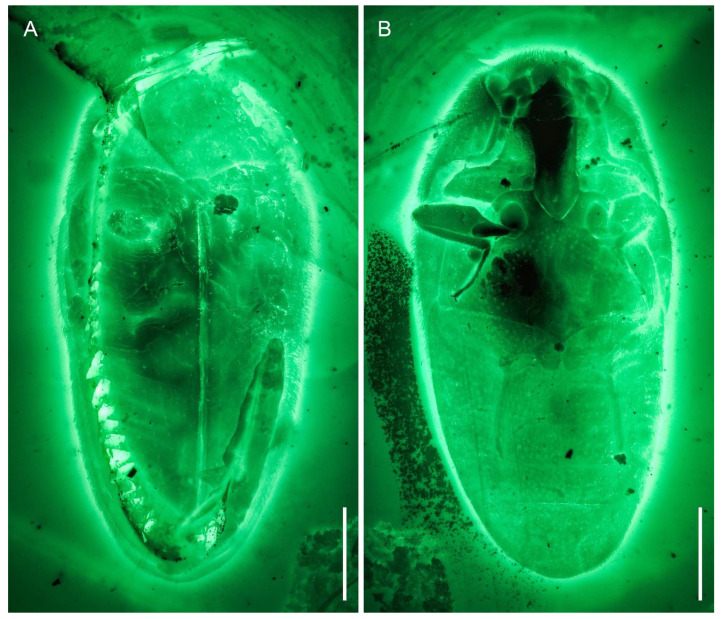
General habitus of *Pseudopactopus robustus* **gen. et sp. nov.**, holotype, NIGP173917, under widefield fluorescence. (**A**) Dorsal view. (**B**) Ventral view. Scale bars: 500 μm.

**Figure 9:**
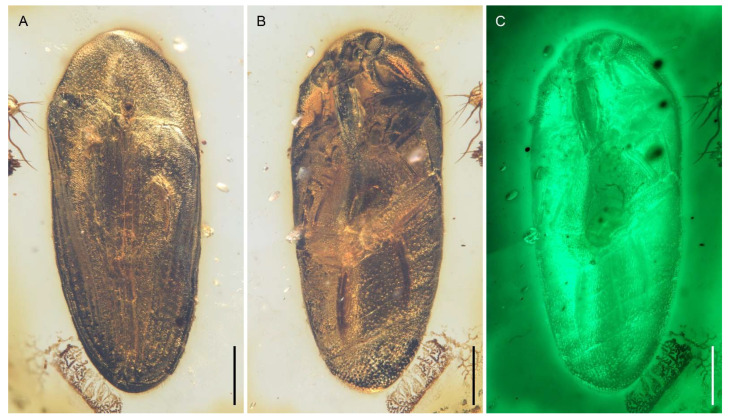
General habitus of *Pseudopactopus robustus* **gen. et sp. nov.**, paratype, NIGP173918 (**A**) Dorsal view, under incident light. (**B**) Ventral view, under incident light. (**C**) Ventral view, under widefield fluorescence. Scale bars: 500 μm.

**Figure 10:**
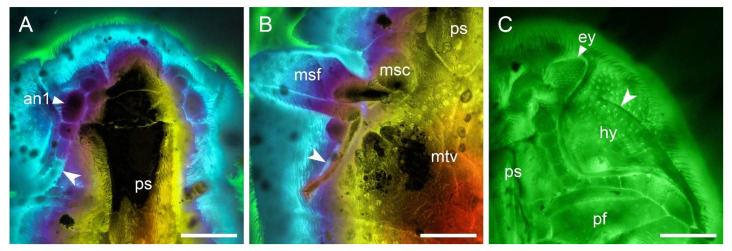
Details of *Pseudopactopus robustus* **gen. et sp. nov.**, under confocal microscopy. (**A**,**B**) Holotype, NIGP173917, with depth colour coding. (**A**) Head and prothorax, ventral view, showing the prothoracic antennal groove (arrowhead). (**B**) Middle leg and metaventrite, ventral view, showing the mesotarsal groove (arrowhead). (**C**) Paratype, NIGP173918, head and prothorax, ventral view, showing the incomplete lateral pronotal carinae (arrowhead). Abbreviations: an1, antennomere 1; ey, compound eye; hy, hypomeron; msc, mesocoxa; msf, mesofemur; mtv, metaventrite; pf, profemur; ps, prosternum. Scale bars: 200 μm.

*Pseudopactopus robustus* Li & Cai **sp. nov.** (Figure 7, Figure 8, Figure 9 and Figure 10).

 

**LSID.** urn:lsid:zoobank.org:act:500CA10A-2F6E-459B-8109-68238492ED8F

**Etymology.** The species is named for its robust appearance.

**Material.** Holotype, NIGP173917, a well-preserved specimen. Paratype, NIGP173918, a well-preserved specimen with body moderately distorted.

**Locality and horizon.** Amber mine located near Noije Bum Village, Tanai Township, Myitkyina District, Kachin State, Myanmar; unnamed horizon, mid-Cretaceous, Upper Albian to Lower Cenomanian [18,19].

**Diagnosis.** As for the genus.

**Description.** Body broad, covered with short hairs. Holotype length 2.9 mm, width 1.4 mm.

Head (Figure 10A) transverse; frons without median carina or other modifications. Eyes large, slightly emarginate, finely facetted, without interfacetal setae. Antennae (Figure 10C) with 11 antennomeres; antennomere 1 large, longer than wide, attached to antennomere 2 apically; antennomere 2 subquadrate, shorter and narrower than scape; antennomere 3 longer and thinner than antennomere 2; terminal three antennomeres enlarged, forming a club.

Pronotal disc about 2.2 times as wide as long along the middle; sides not sinuate, converging anteriorly; posterior angles strongly acute and produced posteriorly; posterior edge bisinuate, with an additional slight emargination in the middle fitting against scutellum; lateral pronotal carinae incomplete (Figure 10C). Scutellum subpentagonal, rounded. Elytra widest behind the base, about 1.6 times as long as wide combined; elytral surface with small to medium-sized punctures on the striae.

Prosternum subtrapezoidal; prosternal carinae almost parallel; prosternal process slightly narrowed in middle, apically acute, fitting into mesoventral cavity. Protibiae simple. Metaventrite without discrimen; mesotarsal grooves well-developed (Figure 10B). Mesocoxae round; metacoxal plates medially extending posteriorly. Tarsal formula 5-5-5; pretarsal claws simple.

Abdomen with five connate ventrites; ventrite 5 twice as long as ventrite 4; metatarsal grooves well-developed, merely extending to the hind margin of ventrite 3.

## 4. Discussion

The three throscid species presented in this paper, together with two previous reported species, show a high degree of morphological variation of Throscidae in Burmese amber. Specialized throscids have also been reported in Lower Cretaceous Lebanese amber [2]. Compared to their extant relatives, these Mesozoic throscids seem to be morphologically more diverse. There are only four known extant genera in Throscidae, while six genera have been established based on Cretaceous fossils. An identification key to genera in Throscidae is updated in light of the new specimens discovered from Burmese amber (Appendix A). Many interesting features found in Mesozoic throscids have hitherto been unknown in Recent Throscidae (e.g., deep cavities on head in *Captopus* **gen. nov.**, small and very narrow eyes in *Potergosoma*, nonparallel prosternal carinae in *Potergosoma* and *Trixagosoma*). The divergence between Throscidae and the group of Eucnemidae + Cerophytidae has been dated to Middle Jurassic, approximately 165 Ma [12]. Therefore it is reasonable to expect that Throscidae might have already been highly diversified in late Mesozoic. We hope further findings on Mesozoic throscid fossils could help us understand the early diversification of this family.

Despite the wide distribution of the family, throscids are poorly studied and their taxonomy is not well resolved. Though the monophyly of Throscidae has been supported by recent molecular phylogenetic analyses [9,23], no molecular-based analysis has focused on the inter-generic relationships within Throscidae. A morphology-based phylogenetic analysis of both extant and extinct throscids was performed by Muona [3]. Although two of three equally shortest trees he obtained from the parsimony analysis showed *Aulonothroscus* as non-monophyletic, he concluded it was reasonable to opt for the tree supporting a monophyletic *Aulonothroscus*. However, later Li et al. [13] performed an analysis under implied weights with a slightly modified dataset, in which *Aulonothroscus* appeared to be paraphyletic in the majority-rule consensus tree.

The generic assignment of some species by Muona [3] was also dubious. For example, a throscid from Eocene Baltic amber, *Trixagus parvulus* Muona, was assigned to genus *Trixagus*. However, the most important diagnostic feature for *Trixagus*, the vestigial tarsal grooves, is not visible on the specimen: the position for possible tarsal groove is concealed by its femur. The club-forming antennomeres in *T. parvulus* seems to be rather symmetrical and moniliform (Figure 16 in [3]), whereas in *Trixagus* they should be asymmetrically expanded and serratiform. Besides, we need to be cautious when evaluating the systematic position of insects in preserved in amber, especially when the specimen is not well-preserved. A failure in identifying a certain structure in an amber specimen does not necessarily mean that it is absent [24]. Various decomposition and distortion processes may lead to the misinterpretation of morphological characters. Further better preserved fossils could be helpful for clarifying the accurate position of these species.

## 5. Conclusions

Our discovery of *Captopus depressiceps* **gen. et sp. nov.**, *Electrothroscus yanpingae* **gen. et sp. nov.** and *Pseudopactopus* *robustus* **gen. et sp. nov.** in the mid-Cretaceous Burmese amber greatly extends the Mesozoic diversity of Throscidae. These newly discovered species imply a high degree of morphological disparity for this family in the Cretaceous. Further phylogenetic analysis with incorporation of both molecular data and fossil taxa would be helpful for elucidating the early evolutionary history of Throscidae.

## Data Availability

The original series of confocal slices are available on Zenodo repository (doi:10.5281/zenodo.3994927).

## Appendix A. Key to Genera of Throscidae (Modified from Muona, 2019)

**1.** Metathorax and abdomen without tarsal grooves; body elongate	2
**1′.** At least metathorax with tarsal grooves	5
**2.** Head without median carina	3
**2′.** Head with median carina	4
**3.** Prosternum with nonparallel prosternal carinae	*Pseudothroscus* Muona
**3′.** Prosternum with subparallel prosternal carinae	*Electrothroscus* gen. nov.
**4.** Antennae with 4 enlarged apical antennomeres	*Jaira* Muona
**4′.** Antennae with 3 enlarged apical antennomeres	*Potergosoma* Kovalev & Kirejtshuk
**5.** Abdomen with tarsal grooves	6
**5′.** Abdomen at most with slight impressions for tarsi	11
**6.** Head with median carina	7
**6′.** Head without median carina	8
**7.** Metaventrite simple	*Potergus* Bonvouloir
**7′.** Metaventrite medially widely keeled, anteriorly deeply bifid	*Rhomboaspis* Kovalev & Kirejtshuk
**8.** Supraocular ridges strong, well developed	*Tyrannothroscus* Muona
**8′.** Supraocular ridges not present	9
**9.** Frons with a pair of deep cavities	*Captopus* gen. nov.
**9′.** Frons simple	10
**10.** Protibiae apically enlarged, with tarsal grooves	*Pactopus* LeConte
**10′.** Protibiae simple	*Pseudopactopus* gen. nov.
**11.** Prosternum with nonparallel prosternal carinae	*Trixagosoma* Li et al.
**11′.** Prosternum with subparallel prosternal carinae	12
**12.** Metaventrite with vestigial tarsal grooves; median lobe longer than parameres	*Triagus* Kugelann
**12′.** Metaventrite with deep, long tarsal grooves; median lobe shorter than parameres	*Aulonothroscus* Horn
